# Many ways to skin a cat: psychometric methods options illustrated

**DOI:** 10.1186/s41687-019-0133-2

**Published:** 2019-07-30

**Authors:** Donald L. Patrick

**Affiliations:** Department of Health Services, University of Washington, Seattle, WA 98195 USA

**Keywords:** Psychometrics, Classical test theory, Item response theory, Rasch measurement theory, Patient-reported outcomes

## Abstract

**Background:**

The three articles in this issue from members of the Psychometric Special Interest Group (SIG) of the International Society for Quality of Life Research (ISOQOL) examine three different psychometric techniques researchers use to analyze item and scale properties of a patient-reported outcome (PRO) instrument. The articles illustrate their respective strengths and weaknesses.

**Main text:**

Many published articles use one of the three methodologies analyzed by the authors and the reader should have a basic familiarity with the assumptions, approaches, and statistical techniques behind each analysis. These three papers shed light on some of the conundrums facing developers and users of PRO measures and data regarding what method and instruments to use. These papers have used a dataset on depressive symptoms to show that no attempt to measure such a complex feeling domain as depressed mood can cover the entire spectrum of the experience.

**Conclusions:**

As a group, these three papers will help readers evaluate published articles on instruments using one or more approaches as well as providing general education on these statistical methods in application.

## Background

The three articles in this issue from members of the Psychometric Special Interest Group (SIG) of the International Society for Quality of Life Research (ISOQOL) illustrate well the old English proverb stating that a problem generally has more than one solution. These papers were intended to show that different psychometric techniques used to analyze item and scale properties of a patient-reported outcome (PRO) instrument all have respective strengths and weaknesses. Many of the techniques are inherent in the theory and methods behind each approach. Authors took data from the Emotional Distress – Depression Item Bank version 1.0 and conducted analyses for preparing these papers. The *Patient-Reported Outcomes Measurement Information System* ***(***PROMIS®) measure and analytic data set were available in the public domain, the instruments have been widely used, and thus it lends itself well to illustrating methods using item banks, long and short forms, and items chosen to reflect a single construct.

## Main text

Psychometrics is the disciplinary home of a set of statistical models and methods developed primarily to summarize, describe, and draw inferences from empirical data [1]. These three papers use methods arising from the three major approaches within psychometrics of psychological scaling, factor analysis, and test theory. These psychometric methods have been in use for decades by other disciplines and by health outcome researchers for analyzing PRO data more recently. Many published articles in this journal use one of these methods and expect the reader to have basic familiarity with the assumptions, approaches, and statistical techniques behind each analysis. Although general treatments of the approaches exist [2, 3], the opportunity made possible in this issue to showcase several different methods in direct application to PROs all in one place, is rare.

These three papers shed light on some of the conundrums facing developers and users of PRO measures and data. For example, users ask, “Do I use a long-form or a short-form of an instrument? What are the differences?” In addition, users may ask, “Will this measure apply to my context of use and target population for assessment?” Developers choose one or more of these methods for evaluating the measurement properties of an instrument and thus ask the question, “What method or methods should I use?” A single paper on each method cannot answer these questions comprehensively. Many theoretical and value judgements are not answered by statistical methodology alone, such as selection of the concept being measured, the comprehensiveness and participant understanding of the items, the use of different recall periods and response options, and the interpretation of scores. These papers reveal the bare bones of the analytic techniques for evaluating measurement properties, however, and it is our hope that, as a group, they will help the readers in evaluating published articles on instruments using one or more approaches as well as providing general education on these statistical methods in application.

The three papers in this volume used data from 51 items of the PROMIS depression bank completed by 825 participants [4]. A sample of 925 individuals completed the computerized PROMIS depression items and 11 demographic items (778 from YouGovPolimetrix and 47 from four PROMIS sites. One-hundred individuals were deleted because they had a mean response time of less than 1 s or 10 or more items in a row where response time was less than 1 s. The final sample size was 825 individuals, one of whom did not report a gender. Table 1 contains the sociodemographic characteristics of the dataset, completed separate for men and women, since there are known differences by gender in reporting of depressive symptoms and function [5], The sample data, collected via web survey and clinic participation, cover a wide range of ages, ethnic background, income, and education in addition to gender. This dataset was sufficiently varied to add credence to the psychometric analyses conducted by the three methods groups.

**Table 1:** Sociodemographics of the PROMIS® Wave 1 Cohort

	Total Sample (*N* = 825)^§^ n (%)	Men (*N* = 400) n (%)	Women (*N* = 424) n (%)	χ2 for gender
Age				5.84
18–34	217 (26%)	102 (25%)	115 (27%)	
35–64	380 (46%)	172 (43%)	207 (49%)	
65+	228 (28%)	126 (32%)	102 (24%)	
Ethnicity				0.06
Hispanic	77 (9%)	38 (10%)	38 (9%)	
Race				10.28
Caucasian	652 (79%)	328 (82%)	324 (76%)	
African American	83 (10%)	35 (9%)	47 (11%)	
Other	90 (11%)	37 (9%)	53 (13%)	
Education				1.10
≤ High School	184 (22%)	89 (22%)	94 (22%)	
Some College	385 (47%)	180 (45%)	205 (48%)	
≥ College	255 (31%)	130 (33%)	125 (29%)	
Marital Status				11.63**
Never Married	136 (16%)	78 (20%)	58 (14%)	
Married/ Partner	523 (63%)	258 (65%)	265 (63%)	
Separated/Divorced	101 (12%)	43 (11%)	58 (14%)	
Widowed	63 (8%)	21 (5%)	42 (10%)	
Occupation				99.56**
Homemaker	51 (6%)	2 (0.5%)	49 (12%)	
Unemployed	28 (3%)	17 (4%)	11 (3%)	
Retired	221 (27%)	127 (32%)	94 (22%)	
Disability	30 (4%)	11 (3%)	18 (4%)	
Leave of Absence	2 (0.2%)	1 (0.25%)	1 (0.24%)	
Full-time employed	318 (39%)	181 (45%)	137 (32%)	
Part-time employed	55 (7%)	20 (5%)	35 (8%)	
Full-time student	25 (3%)	6 (2%)	19 (5%)	
Income				10.00*
< $20,000	85 (10%)	32 (8%)	52 (12%)	
$20,000 - $49,999	292 (35%)	129 (32%)	163 (38%)	
52 (12%)$50,000 - $99,999	320 (39%)	172 (43%)	148 (35%)	
≥ $100,000	105 (13%)	55 (14%)	50 (12%)	
Source				0.50
Web Survey	778 (94%)	380 (95%)	398 (94%)	
Clinical Site	47 (6%)	20 (5%)	26 (6%)	

*p < .05

**p < .01

§Note: one individual did not report gender

*Depression*, or more specifically *depressive mood*, is an excellent concept or construct for illustration. The concept is widely measured and depression or depressed mood constitute a well-demonstrated and significant burden on people who experience feeling *sad, anxious, empty* or one of the many other descriptors or feelings at least a quarter of people have in a lifetime to a significant degree [6]. All three papers, however, point out that no attempt to measure such a complex feeling domain as depression can cover the entire spectrum of the experience, from the most clinically defined population to the most general population made up of members with countless differences, such as age, gender, ethnicity, and coexisting conditions. The continuum of depressive mood stretches from a low level, sometimes characterized as demoralization and not depression, to a high level, sometimes characterized as despair with suicidal thoughts [7].

Testing the depression items at different levels of the depressive feeling’s continuum helps judge how well the instrument is assessing the construct for the target population in mind by the user.

Technology also makes these papers possible. Each paper uses and references a particular statistical package. Though it is theoretically possible to use these methodological approaches with multiple statistical software, the papers illustrate our dependence on package software for implementing analyses and promulgating the use of a particular method. The software also encourages standardization of analyses that improves comparability of results.

The paper on classical test theory applications (Nolte, S., Coon, C., Hudgens, S., & Verdam, M. G. E: Psychometric evaluation of the PROMIS® depression item Bank: An illustration of classical test theory methods, Submitted) is notable for addressing the issue of content validity head on, an important part of validation, though it is often treated as a separate characteristic of measurement. The paper does not and cannot address the need for content validity in different populations, though the PROMIS depression bank has been tested across the general population in the US. Nevertheless, attention to content validity remains important for different populations as illustrated in a study where items in the emotional distress domain were noted missing from the bank for persons who were HIV-positive [8].

Item response theory (IRT) is a statistical modeling paradigm that aims to find the best measurement model to fit the observed data. The paper that showcases IRT methods (Stover, A. M., McLeod, L. M., Langer, M. M., Chen, W.-H., & Reeve, B. B: State of the psychometric methods: Patient-reported outcome measure development and refinement using item response theory, Submitted) illustrates the strength of subgroup analyses and the flexibility of IRT in analyzing psychometric properties. Differential item functioning analyses can be used to detect items performing differently in subgroups, for example, men and women where we know responses to questions on emotional distress may differ by gender [9]. Although the paper points out the need for large sample sizes in calibration studies, considerable advantages are noted, including item-characteristic curves, the development of computer-adapted testing applications, and considerable flexibility in approach.

The paper illustrating Rasch measurement theory (RMT) (Cleanthous, S., Barbic, S., Smith, S., & Regnault, A: Psychometric performance of the PROMIS® depression item Bank: A comparison of the 28- and 51-item versions using Rasch measurement theory, Submitted) brings out the focus of the approach on targeting, response thresholds, and item interdependency. Though similar in approach to IRT, RMT aims to evaluate the extent to which a set of items conforms to the requirement of the Rasch model, and thus the instrument and the set of items are “hypothesizes” about the appropriate content to meet requirements of the method. Also by identifying items or measure characteristics that do not fit the RMT model, the approach encourages iterative development of item content and appraising the qualitative and quantitative evidence to further understand the disparity between expected and observed scores of an evaluated scale. In doing so, the paper makes recommendations for improvement in an already-strong instrument.

None of the papers here address the issue of the ability of the PROMIS depression bank and measures to detect change and the ease of interpretation of that change as the provided dataset did not permit such analyses. This stands as a caution to readers not to make assumptions regarding the suitability of any scale based on one analysis or data collection. Indeed, while the reported results indicate the psychometric soundness of this measure, it is imperative that readers not over-generalize this information and recognize that many important measurement properties of a measure, such as its applicability to other contexts of use, including clinical trials and repeated observational studies, require additional consideration.

All three papers support the strong statistical performance of the PROMIS depression measures. The differences, closely associated with the assumptions and specific methods of the approach, provide the strengths and potential weaknesses of each method. Readers of these articles will benefit greatly from following them closely with particular attention to the software used in producing the analyses and in the conclusions based on the objectives of the analyses and presentations of results.

## Conclusions

Adherents of different psychometric approaches tend to be proponents of the particular method used, sometimes with ferocity and disciple-like emotion. I commend the authors of these papers for tackling what has been a sometimes-contentious debate about different strengths and weaknesses of the methods using transparency and rigor. The interests of the field of health outcome measurement are best served with open discussion of methods. We still have much to share, learn about, and debate in our chosen field of patient-centered outcomes research.

## Data Availability

The datasets mentioned in this article are available from the PROMIS Health Organization, http://www.healthmeasures.net/explore-measurement-systems/promis

## Abbreviations

IRT: Item response theory
ISOQOL: International Society for Quality of Life Research
PRO: Patient-reported outcomes
PROMIS: Patient-reported outcomes measurement information system
RMT: Rasch measurement theory
